# Mendelian randomization analysis reveals causal effects of food intakes on inflammatory bowel disease risk

**DOI:** 10.3389/fimmu.2022.911631

**Published:** 2022-09-22

**Authors:** Bingxia Chen, Zemin Han, Lanlan Geng

**Affiliations:** ^1^ Guangzhou Women and Children’s Medical Center, Guangzhou Medical University, Guangzhou, China; ^2^ Nanfang Hospital, Southern Medical University, Guangzhou, China

**Keywords:** Mendelian randomization analysis, causal effects, food intakes, risk factors, inflammatory bowel disease

## Abstract

Traditional observational studies have indicated a link between specific food intakes and inflammatory bowel disease (IBD), but the nature of such links remains unknown. We sought to assess the potential causal relationship between food intakes and IBD risk using Mendelian randomization methods. This study used summary statistics data from large-scale genome-wide association studies (GWAS) on food intakes, Crohn’s disease (CD), and ulcerative colitis (UC). In the primary analysis, we used the inverse variance-weighted method to determine whether specific food was causal for CD and UC. In addition, we also ran four other Mendelian randomization methods, including MR Egger, weighted median, maximum likelihood, and weighted mode as a complement. The primary analysis showed that high consumption of poultry (OR, 3.696; 95% CI, 1.056–12.937; *p* = 0.041) and cereal (OR, 2.449; 95% CI, 1.094–5.482; *p* = 0.029) had a significant causal association with CD, while high oily fish intake level was found to be statistically significantly associated with the risk of UC (OR, 1.482; 95% CI, 1.002–2.194; *p* = 0.049). This MR study provides evidence of a potential causal link between certain food intake and CD and UC.

## Introduction

Inflammatory bowel disease (IBD), including Crohn’s disease (CD) and ulcerative colitis (UC), is a multifactorial disease characterized by a deregulated immune response to environmental and microbial components on a genetic susceptibility background (1). While several environmental factors participate in the pathogenesis and progression of IBD, the role of diet has attracted considerable attention. Although the exact mechanism remains uncertain, it has been proposed that certain food intake may modify the risk of IBD through its impact on host immunity system, gut barrier, and gut microbiome, all of which are critical factors in IBD pathogenesis (2–6). Many food risk factors have been established to be associated with IBD pathogenesis, especially the components of a Western diet, which is known to be high in fat, n-6 polyunsaturated fatty acids (PUFAs), and red and processed meat, and low in fruits and vegetables (7).

While a few studies have identified some food risk factors for IBD progression, insufficient evidence supports their causal roles in IBD incidence. Some cross-sectional studies were conducted to determine the diet responsible for IBD incidence. These observations, however, might be confounded by unidentified factors and therefore contradict the causality of the associations. RCTs are the gold standard for determining a causal relationship (8, 9). However, due to ethical constraints, an RCT is difficult to implement in most cases. Mendelian randomization (MR) analysis can help overcome these limitations. In MR analysis, genetic variants such as single-nucleotide polymorphisms (SNPs) are used as instrumental variables (IVs) to estimate the causal associations between an exposure and an outcome (10). Since genetic variation is inherited from parents and remains unchanged after birth, the association between genetic variation and outcome is reasonable. MR analysis relies on three critical assumptions: (i) IVs are strongly associated with exposure; (ii) IVs should be independent of confounders of exposure and outcome; and (iii) IV–outcome association is only mediated *via* exposure (10).

Understanding the exact role of foods in IBD risk may be helpful to develop more effective prevention, prediction, and treatment strategies for essential conditions. Therefore, we applied the MR method to analyze the causal relationships between food intakes and two IBD subtypes, CD and UC.

## Methods

### Data sources

#### Genome-wide association studies of food intakes

**Figure 1:**
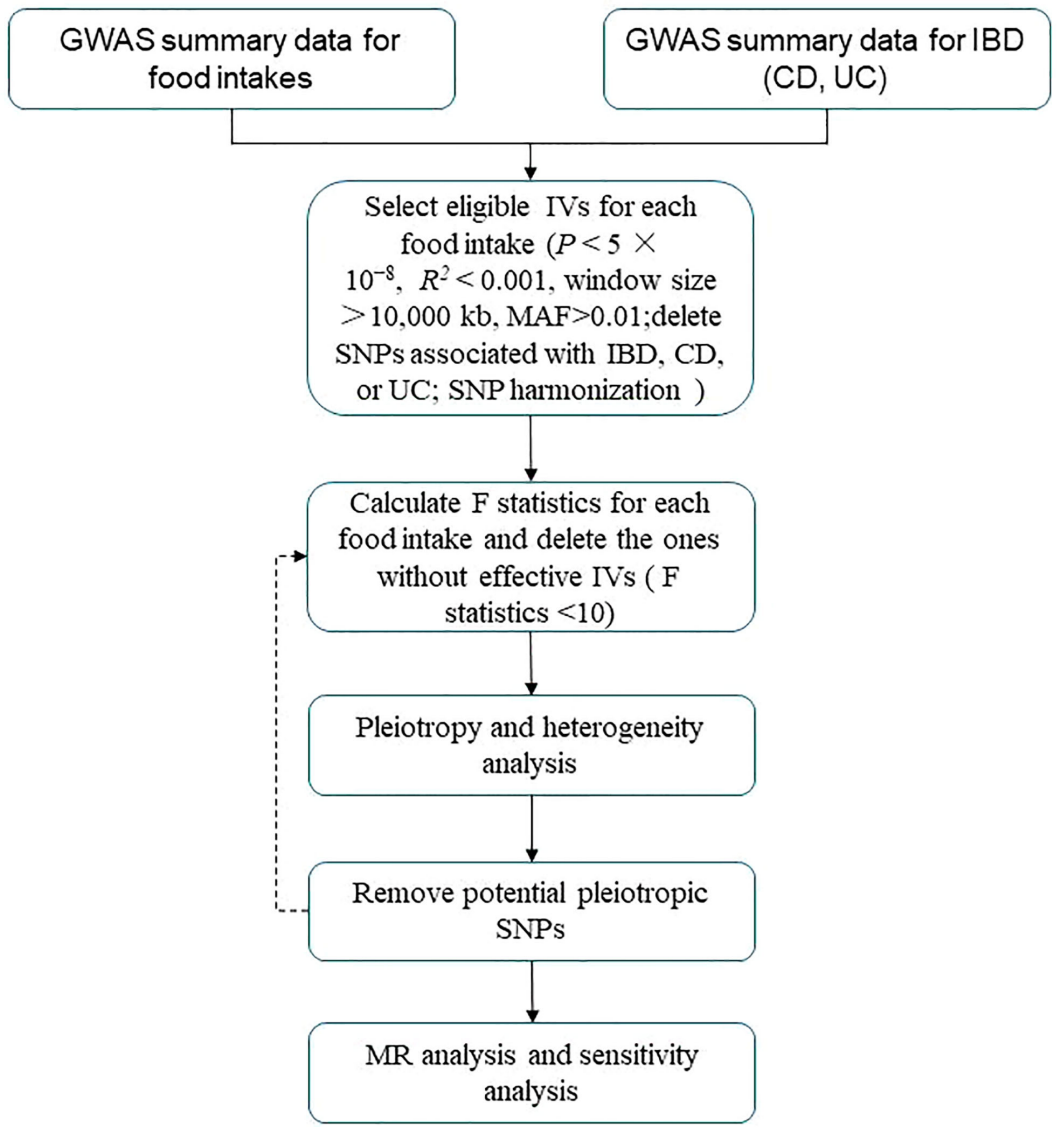
Flowchart of MR analysis in this study. IBD, inflammatory bowel disease; CD, Crohn’s disease; UC, ulcerative colitis; MAF, minor allele frequency; IVs, instrumental variables; SNPs, single-nucleotide polymorphisms; MR analysis, Mendelian randomization analysis.

A flowchart describes the study design briefly (**Figure 1**). For summary statistics for food intakes, we used data from the UK Biobank (UKB). The UKB project is a large, prospective cohort study with about 500,000 participants from the United Kingdom (11).

#### Genome-wide association studies of CD and UC

For CD and UC, we used publicly available meta-analysis GWAS (12). GWAS of CD (ebi-a-GCST004132) included 12,194 cases and 28,072 control subjects, and GWAS of UC (ebi-a-GCST004133) included 12,366 cases and 33,609 control subjects.

#### Genetic IV selection

We selected eligible genetic IVs using a series of quality control criteria based on the GWAS summary food data. First, we used independent genetic variants significantly associated (*p* < 5 × 10^−8^) for each instrument with each exposure. Then, we performed the clumping procedure with *R* ^2^ < 0.001 and a window size >10,000 kb to avoid linkage disequilibrium (LD) (13). Third, we eliminated SNPs with a minor allele frequency (MAF) of less than 0.01. Fourth, to prevent potential pleiotropic effects for the instruments, we used Phenoscanner, a database that includes genotype–phenotype associations (14). We searched for each SNP included as an instrument in our analysis and removed SNPs associated with IBD, CD, or UC. SNP harmonization was also performed to rectify the orientation of the alleles (15).

### Evaluation of the strength of the genetic instruments

To assess the strength of genetic instruments for each food intake, we calculated the proportion of variance explained (*R* ^2^) and *F* statistics for all SNPs. IVs (*F* statistics < 10) were considered weak instruments, and the exposure would be excluded from MR analysis (16).

### Pleiotropy, heterogeneity, and sensitivity analysis

MR Egger regression was used to assess the possibility of horizontal pleiotropy, as indicated by the intercept (17). In the presence of pleiotropy (*p* ≤ 0.05), MR Pleiotropy REsidual Sum and Outlier (MR-PRESSO) test was conducted using MR-PRESSO package in R, and SNP with the smallest pleiotropy *p*-value was removed (18). In addition, we used the inverse-variance weighted (IVW) approach and MR Egger regression to identify heterogeneity, which was quantified using Cochran’s *Q* statistic. In addition, we conducted the leave-one-out analysis to identify the stability of results.

### MR analysis

In this study, we used IVW two-sample MR as our primary analysis (19) to estimate the causal effects of exposures on the risk of CD and UC. The IVW method assumes that all variants are valid IVs, providing the most precise results. In addition, we ran MR Egger, weighted median, maximum likelihood, and weighted mode as a complement. The MR analysis was carried out in R using TwoSampleMR and MendelianRandomization packages (20).

## Results

Overall, 17 kinds of food intake exposures were included in our study, excluding those without effective IVs (i.e., milk intake). The number of SNPs for each food intake ranged from 6 to 48 after a series of quality control steps (**Tables 1**, **2**). The *F* statistic values were more than the empirical threshold of 10, suggesting that all SNPs had sufficient validity.

**Table 1:** Summary of modifiable risk factors for CD.

Exposure	GWAS data	Nsnp	sample	R2	F
Alcohol intake frequency	ukb-a-25	29	336965	0.00159	18.5018
Beef intake	ukb-b-2862	10	461053	0.00032	14.6281
Bread intake	ukb-b-11348	24	452236	0.00096	18.0272
Cereal intake	ukb-b-15926	29	441,640	0.00118	17.9189
Cheese intake	ukb-b-1489	48	451,486	0.00145	13.688
Coffee intake	ukb-b-5237	32	428860	0.00236	31.7542
Cooked vegetable intake	ukb-b-8089	12	448651	0.00046	17.2863
Dried fruit intake	ukb-b-16576	30	421,764	0.00112	15.7614
Fresh fruit intake	ukb-b-3881	38	446462	0.00166	19.5377
Lamb intake	ukb-b-14179	25	460006	0.00082	15.0406
Non-oily fish intake	ukb-b-17627	9	460,880	0.00036	18.6157
Oily fish intake	ukb-b-2209	42	460,443	0.00163	17.8875
Pork intake	ukb-b-5640	10	460162	0.00035	16.0884
Poultry intake	ukb-b-8006	6	461900	0.00019	14.4174
Processed meat intake	ukb-b-6324	19	461981	0.00062	15.1364
Raw vegetable intake	ukb-b-1996	9	435435	0.00031	14.9826
Tea intake	ukb-b-6066	32	447,485	0.00194	27.1543

F, F statistics; R^2^, phenotype variance explained by genetics.

**Table 2:** Summary of modifiable risk factors for UC.

Exposure	GWAS data	Nsnp	sample	R2	F
Alcohol intake frequency	ukb-a-25	29	336965	0.00159	18.5018
Beef intake	ukb-b-2862	10	461053	0.00032	14.6281
Bread intake	ukb-b-11348	24	452236	0.00096	18.0272
Cereal intake	ukb-b-15926	29	441,640	0.00118	17.9189
Cheese intake	ukb-b-1489	48	451,486	0.00145	13.688
Coffee intake	ukb-b-5237	32	428860	0.00236	31.7542
Cooked vegetable intake	ukb-b-8089	12	448651	0.00046	17.2863
Dried fruit intake	ukb-b-16576	30	421,764	0.00112	15.7614
Fresh fruit intake	ukb-b-3881	38	446462	0.00166	19.5377
Lamb intake	ukb-b-14179	25	460006	0.00082	15.0406
Non-oily fish intake	ukb-b-17627	9	460,880	0.00036	18.6157
Oily fish intake	ukb-b-2209	43	460,443	0.00166	17.8055
Pork intake	ukb-b-5640	10	460162	0.00035	16.0884
Poultry intake	ukb-b-8006	6	461900	0.00019	14.4174
Processed meat intake	ukb-b-6324	19	461981	0.00062	15.1364
Raw vegetable intake	ukb-b-1996	9	435435	0.00031	14.9826
Tea intake	ukb-b-6066	32	447,485	0.00194	27.1543

F, F statistics; R^2^, phenotype variance explained by genetics.

**Figure 2:**
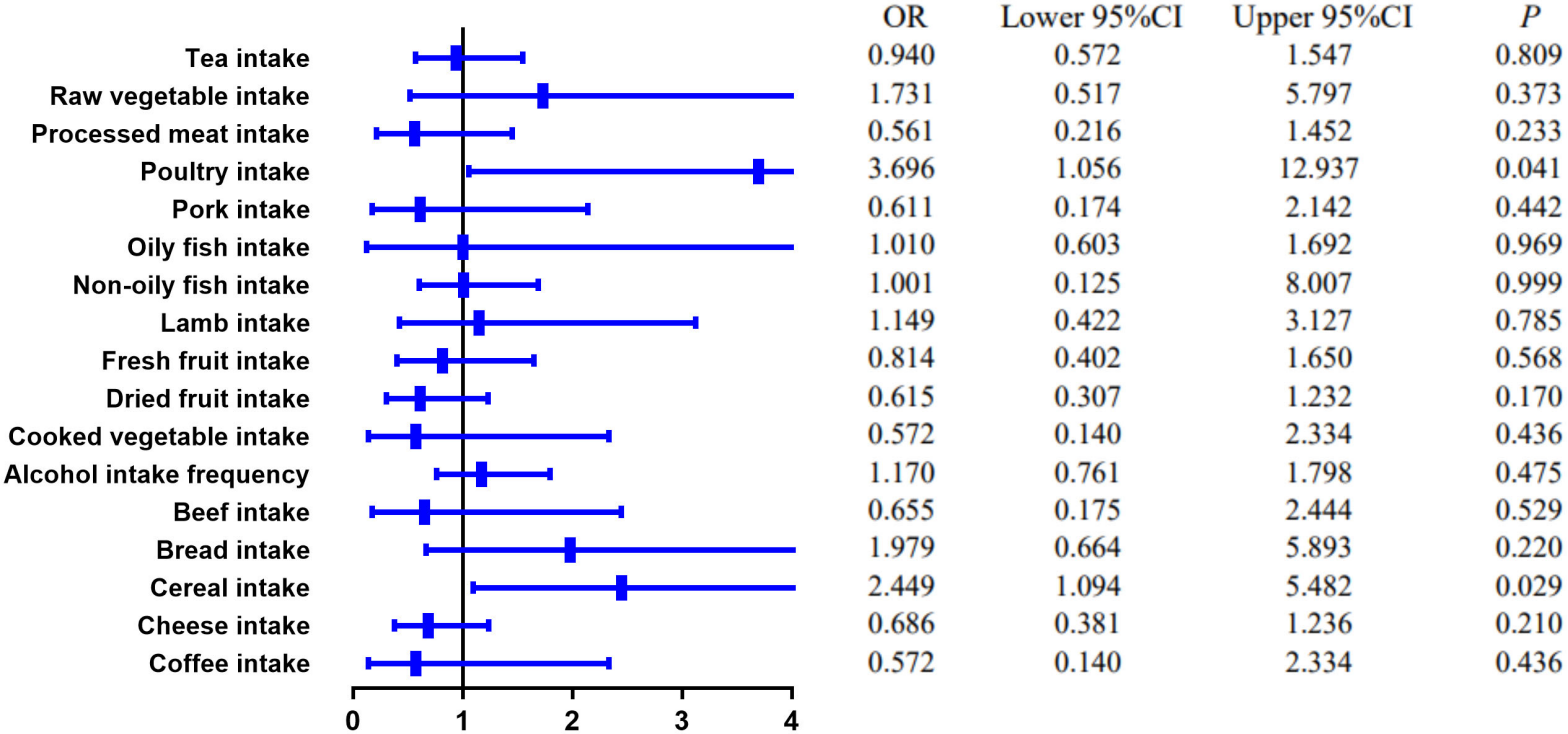
The causal effect of food risk factors on CD based on the IVW method. IVW, inverse-variance weighted; CD, Crohn’s disease.

**Figure 3:**
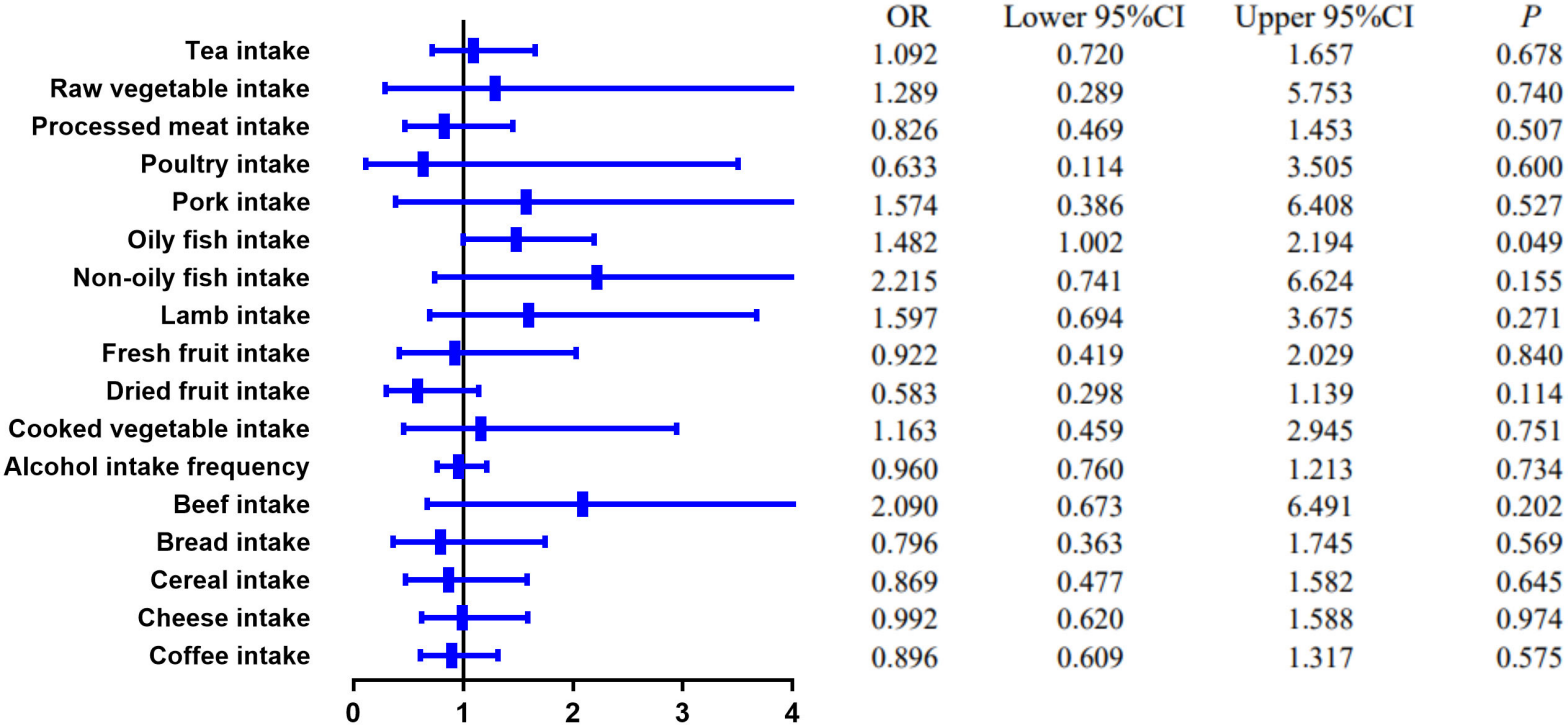
The causal effect of food risk factors on UC based on the IVW method. Abbreviations: IVW, inverse-variance weighted; UC, ulcerative colitis.

MR estimates of different methods are presented in **Tables 3**, **4**. In the primary IVW MR analysis, two causal associations from 17 food intakes to CD were identified, while only one causal association was observed for UC. As shown in **Figures 2**, **3**, We found evidence that increased consumption of poultry was associated with a higher risk of CD (OR, 3.696; 95% CI, 1.056–12.937; *p* = 0.041) but was not associated with UC (OR, 0.633; 95% CI, 0.114–3.505; *p* = 0.600). Higher cereal intake level also increased CD risk (OR, 2.449; 95% CI, 1.094–5.482; *p* = 0.029). On the contrary, genetically predicted oily fish intake level was statistically significantly associated with the risk of UC (OR, 1.482; 95% CI, 1.002–2.194; *p* = 0.049) but not with CD (OR, 1.010; 95% CI, 0.603–1.692; *p* = 0.969). In addition to the above causal associations identified by the IVW test, several intriguing results were identified by other MR tests, including cheese intake based on maximum likelihood method (OR, 0.671; 95% CI, 0.474–0.950; *p* = 0.024) and processed meat intake based on the weighted median method (OR, 0.360; 95% CI, 0.136–0.952; *p* = 0.040), both of which were suggested to be associated with a decreased risk of CD (**Table 3**). Conversely, bread intake based on the weighted median method (OR, 0.332; 95% CI, 0.146–0.752; *p* = 0.008) and dried fruit intake based on the MR Egger method (OR, 0.029; 95% CI, 0.001–0.603; *p* = 0.030) were suggested to be associated with a decreased UC risk (**Table 4**).

**Table 3:** Results of the MR study testing causal association between risk factors and CD.

Analysis	OR	Lower 95%CI	Upper 95%CI	*P*	Horizontal pleiotropy: Egger intercept	Horizontal pleiotropy: *P*	Heterogeneity: Q	Heterogeneity: *P*
**Alcohol intake frequency**								
Inverse variance weighted	1.169647401	0.760988571	1.797760301	0.474894449			142.7444983	2.46E-17
MR Egger	1.15593862	0.574253199	2.326837876	0.687950469	0.000526157	0.966538314	142.7350209	1.05E-17
Weighted median	0.982920368	0.753751511	1.281765191	0.898787879				
Maximum likelihood	1.181986806	0.969840296	1.440539042	0.097604461				
Weighted mode	0.998823095	0.769639652	1.296252828	0.992997648				
**Beef intake**								
Inverse variance weighted	0.654920282	0.175494129	2.444073653	0.528743351			17.02885728	0.048265987
MR Egger	6.851272932	0.010299022	4557.708782	0.577701123	-0.02918774	0.489834944	15.98236563	0.042633211
Weighted median	0.643839295	0.152724063	2.714235266	0.548640484				
Maximum likelihood	0.646566036	0.242329443	1.725121118	0.383789127				
Weighted mode	3.839498843	0.260047095	56.68877536	0.352957322				
**Bread intake**								
Inverse variance weighted	1.978647471	0.664333747	5.893191229	0.220373356			112.0995093	1.07E-13
MR Egger	0.162458241	0.001429798	18.4590313	0.45966761	0.037786467	0.298998447	106.6161849	4.41E-13
Weighted median	1.061414065	0.476670475	2.363477236	0.883978243				
Maximum likelihood	2.043192563	1.20608453	3.461312824	0.007890577				
Weighted mode	0.978319172	0.340335083	2.812253129	0.967896386				
**Cereal intake**								
Inverse variance weighted	2.448873254	1.093913159	5.482135549	0.029382649			78.13927106	1.26E-06
MR Egger	0.826222049	0.028245482	24.16821477	0.912572438	0.015918889	0.52118148	76.93535604	1.09E-06
Weighted median	1.613690292	0.764288777	3.407084383	0.209477226				
Maximum likelihood	2.589344829	1.572245316	4.264415086	0.00018569				
Weighted mode	1.508534323	0.434752502	5.234416809	0.522446126				
**Cheese intake**								
Inverse variance weighted	0.685831348	0.380528367	1.23608298	0.209553962			147.3826261	2.75E-12
MR Egger	0.537858519	0.033974083	8.515072598	0.661936682	0.004026947	0.860585116	147.2827513	1.57E-12
Weighted median	0.677237813	0.394858034	1.161559385	0.156802822				
Maximum likelihood	0.671012053	0.474058762	0.949791905	0.024412055				
Weighted mode	0.966088317	0.317692296	2.937832142	0.951776305				
**Coffee intake**								
Inverse variance weighted	0.682662282	0.409195296	1.138888438	0.14375358			53.38027534	0.007489816
MR Egger	1.220025054	0.454195197	3.27713975	0.696015407	-0.01143769	0.190213896	50.36448599	0.011362878
Weighted median	0.864637558	0.499161407	1.497708146	0.603832776				
Maximum likelihood	0.675640323	0.455439122	1.002307058	0.051349418				
Weighted mode	0.811097338	0.447165517	1.471220092	0.495856147				
**Cooked vegetable intake**								
Inverse variance weighted	0.571785786	0.140083228	2.333890999	0.436006095			24.66645383	0.010198131
MR Egger	0.033336015	6.44E-09	172546.4557	0.675468655	0.029889359	0.724911973	24.34746142	0.006729646
Weighted median	0.292268916	0.070485923	1.211889058	0.090045312				
Maximum likelihood	0.568247635	0.216033509	1.494700407	0.252024459				
Weighted mode	0.250023217	0.024158544	2.587556951	0.269584604				
**Dried fruit intake**								
Inverse variance weighted	0.615304341	0.307418278	1.231544966	0.170148865			47.04758166	0.018410909
MR Egger	0.224767651	0.008201675	6.159778309	0.384386865	0.012339299	0.546753932	46.43047001	0.015721818
Weighted median	0.83355948	0.353232581	1.967036576	0.67770847				
Maximum likelihood	0.602608773	0.345460474	1.051168979	0.074390077				
Weighted mode	0.970658176	0.200630583	4.696080145	0.970718606				
**Fresh fruit intake**								
Inverse variance weighted	0.813916179	0.401507938	1.64992889	0.567928568			48.94698785	0.090431333
MR Egger	1.632089057	0.156408303	17.03051969	0.684664341	-0.006739103	0.54545628	48.44565387	0.080469374
Weighted median	1.716357548	0.661032282	4.456489211	0.267140725				
Maximum likelihood	0.815212336	0.437323812	1.519631757	0.520227301				
Weighted mode	2.123752376	0.463967221	9.721212941	0.33810278				
**Lamb intake**								
Inverse variance weighted	1.149166283	0.422342665	3.126804971	0.785432484			52.32681241	0.000709139
MR Egger	5.268576304	0.078477059	353.7071433	0.446668649	-0.017246051	0.472229933	51.13906726	0.000651619
Weighted median	1.256552995	0.443832079	3.557483796	0.667114082				
Maximum likelihood	1.156211872	0.576538853	2.318709112	0.682661241				
Weighted mode	1.645786546	0.293623491	9.224784239	0.576295982				
**Non-oily fish intake**								
Inverse variance weighted	1.001025076	0.125145745	8.007073662	0.99922943			36.44842618	1.45E-05
MR Egger	0.037940112	1.12E-06	1287.066846	0.558187411	0.04098059	0.549586776	34.50112549	1.39E-05
Weighted median	1.011385888	0.174560653	5.859862455	0.989922164				
Maximum likelihood	1.001089469	0.360585725	2.779311703	0.99833237				
Weighted mode	14.32402576	0.058941784	3481.023152	0.370019788				
**Oily fish intake**								
Inverse variance weighted	1.010209832	0.603125281	1.69205957	0.969208727			76.07911037	0.000713223
MR Egger	1.408535149	0.16947193	11.70678392	0.752851897	-0.005038718	0.752568939	75.88794601	0.000527721
Weighted median	1.541362309	0.865893135	2.743754016	0.141404108				
Maximum likelihood	1.010819232	0.68687898	1.48753354	0.95646407				
Weighted mode	2.406958199	0.74374486	7.789563437	0.150301346				
**Pork intake**								
Inverse variance weighted	0.611217198	0.174401093	2.14211079	0.441649591			11.44569682	0.2463907
MR Egger	0.044283546	7.58E-06	258.8258748	0.501162414	0.026192874	0.565139331	10.95289247	0.204382691
Weighted median	0.606539145	0.129225252	2.84688735	0.526218421				
Maximum likelihood	0.603929827	0.194917394	1.871209284	0.382103021				
Weighted mode	1.078369975	0.08426568	13.8001829	0.955007525				
**Poultry intake**								
Inverse variance weighted	3.696240456	1.056093037	12.9365435	0.040816577			3.965493961	0.554394626
MR Egger	0.000248918	4.06E-19	1.52432E+11	0.657797598	0.104102162	0.609508348	3.659321538	0.454069988
Weighted median	2.327886899	0.488914299	11.08385953	0.288572795				
Maximum likelihood	3.797450458	1.055752067	13.65910656	0.041045459				
Weighted mode	1.97554832	0.281489565	13.86478097	0.523881078				
**Processed meat intake**								
Inverse variance weighted	0.560554205	0.216348782	1.452381724	0.233392724			50.94058035	5.43E-05
MR Egger	0.016445766	0.000250503	1.079680901	0.071250381	0.053851401	0.108671692	43.58995917	0.000393545
Weighted median	0.360348345	0.136368167	0.952208513	0.039515532				
Maximum likelihood	0.569962962	0.316790992	1.025464062	0.060643369				
Weighted mode	0.196607582	0.032098081	1.204263294	0.095565668				
**Raw vegetable intake**								
Inverse variance weighted	1.73146074	0.517146747	5.797109448	0.373243068			8.261610977	0.408341507
MR Egger	10.75657584	0.021916661	5279.267807	0.476879862	-0.019325139	0.573642779	7.870052993	0.34419196
Weighted median	1.905929668	0.375648831	9.670116351	0.436346091				
Maximum likelihood	1.753037449	0.525166816	5.851741204	0.361362317				
Weighted mode	0.966093391	0.098307225	9.49407774	0.977121689				
**Tea intake**								
Inverse variance weighted	0.940489324	0.571833233	1.546814906	0.809016946			73.13197196	2.93E-05
MR Egger	0.705577661	0.234164514	2.126025955	0.540132354	0.006373046	0.570364533	72.33800529	2.34E-05
Weighted median	0.890524492	0.545123898	1.454777296	0.643346379				
Maximum likelihood	0.939417828	0.675566791	1.30631918	0.710257443				
Weighted mode	0.830331203	0.507355126	1.358909907	0.465001237				

**Table 4:** Results of the MR study testing causal association between risk factors and UC.

Analysis	OR	Lower 95%CI	Upper 95%CI	*P*	Horizontal pleiotropy: Egger intercept	Horizontal pleiotropy: *P*	Heterogeneity: Q	Heterogeneity: *P*
**Alcohol intake frequency**								
Inverse variance weighted	0.960248007	0.760082379	1.213126709	0.733776622			41.1857892	0.051635149
MR Egger	0.870756621	0.590471541	1.284087448	0.49095844	0.004184219	0.53912919	40.6038877	0.044938595
Weighted median	0.893173489	0.68398792	1.166334753	0.40664003				
Maximum likelihood	0.959760455	0.790005161	1.165992548	0.679184457				
Weighted mode	0.906090971	0.691078402	1.187999574	0.481421323				
**Beef intake**								
Inverse variance weighted	2.08971835	0.67272235	6.491419201	0.202488462			12.8357884	0.170178529
MR Egger	0.865514015	0.002594981	288.6781755	0.9623321	0.010931152	0.768976796	12.6893222	0.122995545
Weighted median	1.203224862	0.300864509	4.811966928	0.793626202				
Maximum likelihood	2.160150764	0.818945756	5.697875944	0.11961974				
Weighted mode	0.968655764	0.143883377	6.521211871	0.974602207				
**Bread intake**								
Inverse variance weighted	0.79633976	0.363431578	1.744914451	0.56935088			59.20456914	4.97E-05
MR Egger	0.153345443	0.004996258	4.706487583	0.294751062	0.024876184	0.343166767	56.78201626	6.60E-05
Weighted median	0.331651725	0.146262563	0.752023378	0.008234821				
Maximum likelihood	0.799179923	0.482767401	1.322973648	0.383382668				
Weighted mode	0.292600777	0.085471082	1.001686326	0.062553682				
**Cereal intake**								
Inverse variance weighted	0.86870323	0.476918742	1.582335176	0.64547338			44.5670777	0.024394447
MR Egger	0.507493834	0.040642717	6.336928523	0.602792311	0.007877317	0.670625861	44.26398594	0.01941646
Weighted median	0.878907073	0.447085674	1.727806743	0.708193272				
Maximum likelihood	0.864946968	0.533304378	1.40282602	0.556493331				
Weighted mode	0.692471792	0.241686082	1.984049634	0.499432858				
**Cheese intake**								
Inverse variance weighted	0.992125529	0.619882892	1.587901647	0.973717788			96.9417767	2.52E-05
MR Egger	1.111798961	0.12184206	10.14507571	0.92555859	-0.001885371	0.918116069	96.91926185	1.71E-05
Weighted median	0.875180286	0.524780007	1.459545948	0.609397073				
Maximum likelihood	0.992079861	0.7084839	1.389195224	0.963077857				
Weighted mode	0.986653645	0.322328954	3.020161251	0.981319408				
**Coffee intake**								
Inverse variance weighted	0.895537381	0.608756811	1.317418033	0.575326559			31.63882581	0.43438107
MR Egger	1.968836193	0.936007758	4.141328874	0.084264078	-0.015616862	0.021762012	25.77992846	0.68631547
Weighted median	1.26405984	0.724942995	2.204100583	0.408767719				
Maximum likelihood	0.894671694	0.609099401	1.314132697	0.570453671				
Weighted mode	1.315266422	0.769602902	2.247816058	0.323990157				
**Cooked vegetable intake**								
Inverse variance weighted	1.162750658	0.45901774	2.945396166	0.750502303			7.484565454	0.75859546
MR Egger	0.005200763	2.98E-07	90.75920993	0.316105362	0.056857224	0.301057114	6.295299981	0.789873266
Weighted median	0.889136555	0.247041646	3.200123644	0.857285088				
Maximum likelihood	1.166201912	0.456728964	2.977754871	0.747851961				
Weighted mode	0.809933289	0.134585228	4.874174834	0.822151949				
**Dried fruit intake**								
Inverse variance weighted	0.582988554	0.298299772	1.13937618	0.114488952			45.05066688	0.029092239
MR Egger	0.029417951	0.001434985	0.603083585	0.029883024	0.036584528	0.057274734	39.50376068	0.073153933
Weighted median	0.642064307	0.293440539	1.404872605	0.267403159				
Maximum likelihood	0.575472055	0.332343039	0.996464639	0.04853619				
Weighted mode	0.541644202	0.114922834	2.552829848	0.444521069				
**Fresh fruit intake**								
Inverse variance weighted	0.921707183	0.418660439	2.029196102	0.83953786			62.78587591	0.005117006
MR Egger	1.609149166	0.116406346	22.24415697	0.724655302	-0.00539628	0.66509333	62.45535338	0.004048581
Weighted median	0.707068848	0.282145682	1.771944031	0.459598548				
Maximum likelihood	0.917998935	0.495411178	1.701055772	0.785716863				
Weighted mode	0.363220316	0.071275011	1.850985299	0.230585712				
**Lamb intake**								
Inverse variance weighted	1.596607219	0.69368148	3.674820052	0.271300272			37.23079472	0.041493602
MR Egger	2.53098608	0.07349768	87.15772386	0.611966539	-0.005216688	0.795002341	37.11928686	0.031590288
Weighted median	1.649723541	0.585837227	4.645638129	0.34327364				
Maximum likelihood	1.624573147	0.82102678	3.214557642	0.163427125				
Weighted mode	1.661828249	0.234419786	11.78088751	0.615889217				
**Non-oily fish intake**								
Inverse variance weighted	2.214841648	0.740553204	6.62413382	0.154841232			10.43809609	0.235612902
MR Egger	0.015131684	0.000149407	1.532510366	0.118501866	0.06241302	0.067260854	5.757425085	0.568341905
Weighted median	1.25048845	0.348320325	4.489319894	0.731765726				
Maximum likelihood	2.265324724	0.854094121	6.008349641	0.100363264				
Weighted mode	0.930081082	0.191862433	4.508703478	0.930498104				
**Oily fish intake**								
Inverse variance weighted	1.482394299	1.001507108	2.194185983	0.049122493			47.03784999	0.273840038
MR Egger	0.367261456	0.079005868	1.70722732	0.208532831	0.02102955	0.073404418	43.45927354	0.367048836
Weighted median	1.110973272	0.635319533	1.942741479	0.712070699				
Maximum likelihood	1.501367322	1.031370308	2.185542687	0.033902188				
Weighted mode	0.823172572	0.306144699	2.213375195	0.701742697				
**Pork intake**								
Inverse variance weighted	1.573711839	0.386499644	6.407687546	0.526749562			14.74009256	0.098327081
MR Egger	0.000322663	1.10E-07	0.945155904	0.083842859	0.084709306	0.067988915	9.47345866	0.303941398
Weighted median	1.161995553	0.226091384	5.972070404	0.857334701				
Maximum likelihood	1.616430281	0.526494669	4.962722336	0.401421025				
Weighted mode	0.877677245	0.07609348	10.12330285	0.91900186				
**Poultry intake**								
Inverse variance weighted	0.632751142	0.114218061	3.505347601	0.600287511			9.616553531	0.086858328
MR Egger	3.27E-08	1.18E-29	9.04614E+13	0.531391488	0.181828392	0.541535621	8.655194963	0.070320135
Weighted median	0.474914587	0.08982661	2.510880291	0.38080366				
Maximum likelihood	0.617644559	0.173729621	2.195853534	0.456537266				
Weighted mode	0.254485331	0.024928949	2.59789471	0.300451715				
**Processed meat intake**								
Inverse variance weighted	0.825911585	0.469339896	1.453381553	0.507123208			18.53478618	0.420980186
MR Egger	1.584346802	0.111910962	22.42992773	0.737780892	-0.009951475	0.627878754	18.27286259	0.371825679
Weighted median	0.927763989	0.430362922	2.000046878	0.848280491				
Maximum likelihood	0.827088746	0.470409997	1.454211854	0.509651958				
Weighted mode	1.047580936	0.284305205	3.860027174	0.945077071				
**Raw vegetable intake**								
Inverse variance weighted	1.288586424	0.288628987	5.752904398	0.739775697			12.97118688	0.112844833
MR Egger	0.050286437	3.33E-05	75.8750762	0.449620819	0.034430372	0.404086986	11.65828902	0.112371136
Weighted median	1.696732148	0.315010206	9.139068901	0.53828427				
Maximum likelihood	1.300514873	0.392751459	4.306384849	0.667101775				
Weighted mode	1.73835638	0.135448067	22.31026965	0.682279121				
**Tea intake**								
Inverse variance weighted	1.092154285	0.720017415	1.65662796	0.67835992			53.35359083	0.007539068
MR Egger	1.200798319	0.477468781	3.019918076	0.700102019	-0.002116573	0.822091289	53.26223943	0.005549248
Weighted median	1.242149121	0.780737408	1.976252737	0.360052069				
Maximum likelihood	1.095314013	0.794058595	1.510861786	0.579041763				
Weighted mode	1.207024021	0.757574296	1.923120932	0.434524581				

The scatter plots, forest plots, funnel plots, and leave-one-out plots for CD and UC are displayed in **Figures S1A–Q**, **S2A–Q**, **S3A–Q**, **S4A–Q**, **S5A–Q**, **S6A–Q**, **S7A–Q**, and **S8A–Q**.

## Discussion

The etiology of IBD is complex, involving immune imbalance, like dysregulated IL-23/Th17, alteration of microbiome, and infection (1, 21). There is mounting evidence that certain foods may increase or decrease IBD risk in susceptible individuals (2, 4, 22–26). MR analysis was conducted to evaluate the potential causality between food intakes and IBD in this study, which uses random allocation of alleles to replicate the randomization process in double-blind clinical trials. Using large-scale summary statistics from food intake GWAS and CD, UC GWAS, we identified specific food intake that might be causally associated with CD and UC risk.

High red meat intake is one of the features of the Western diet, which is believed to be a risk factor for IBD. Peters et al. found that the “carnivorous” dietary pattern, which consists of high consumption of red meat, poultry, and processed meat, was associated with UC development (OR: 1.11, 95% CI, 1.01–1.22, *p* = 0.024) but not with CD (OR: 0.99, 95% CI, 0.86–1.33, *p* = 0.853) in a prospective population-based cohort (22). Animal studies have indicated that iron, sulfur, and fats are risk factors for colitis and ileitis, which are found in high concentrations in meats (27, 28). Hydrogen sulfide (H_2_S) has been demonstrated to have detrimental inflammatory effects on the colon (29). However, our MR study did not find any association between red meat intake (beef intake, lamb intake, and pork intake) and IBD risk but discovered a causal association between poultry intake and CD risk. Based on another large, multinational, prospective cohort study involving 116,087 participants from 21 countries, intakes of red meat and white meat were not associated with incident IBD (30).

Despite previous evidence from human studies supporting a possible link between processed meat consumption and IBD, the conclusion is inconsistent. It was reported that higher processed meat consumption was associated with a higher risk of IBD in Narula et al.’s study (30). However, in another prospective cohort study of three national cohorts of American health professionals, which included 245,112 participants, the author found that although higher ultra-processed food intake was associated with an increased risk of incident CD, meat products were not related to the risk of CD (23). In contrast, ultra-processed bread and other processed foods showed positive associations with CD risk (23). The conclusion is controversial because when people cut back on processed meat in their diet, they must replace it with something else. Participants in different studies may replace processed meat with different foods, affecting CD risk. Through MR analysis, our study suggested that there might be an inverse causal relationship between processed meat consumption and CD risk.

Another feature of the Western diet is a low intake of fruits and vegetables. Diets high in fruits and vegetables were found to be inversely related to CD in a large prospective cohort study (26). In addition, a nested matched case–control study using a large European Prospective Investigation into Cancer and Nutrition (EPIC) prospective database found that low vegetable intake was associated with an increased risk of UC (31). One explanation of fruit and vegetable’s beneficial role in IBD is high fiber. Because fiber reduces intestinal transit times, potential toxic exposures have less time to contact the intestinal wall. In addition, fiber can be converted into short-chain fatty acids (SCFAs), such as butyrate, which enhances mucus and antimicrobial peptide secretion, and modulates intestinal inflammation by suppressing pro-inflammatory mediators (32, 33). Fiber may also help maintain the intestinal barrier by reducing pathogen translocation across Peyer’s patches and colonic lymphoid follicles (34). Despite a few researchers reporting some protective effects and all of these proposed protective mechanisms, studies of fiber and disease onset and clinical relapse of IBD did not find any consistent effects (4, 35, 36). Narula and his colleagues reported that intake of fruit and vegetables was not associated with incident IBD (30). Based on the MR Egger method, dried fruit intake might be associated with a decreased UC risk in our study, while no relationship was found between fruit or vegetable intake and CD in our study.

Our MR analysis also indicated that high oily fish intake level might increase the risk of UC. Interestingly, in a meta-analysis, increased fish intake was a protective factor for CD in Western countries, but a risk factor for UC in Eastern countries (37). In a prospective cohort study consisting of 67581 women living in France, high consumption of meat or fish but not dairy products was found to be associated with IBD risk among sources of animal protein (38).

In addition, we found that cereal intake may increase the risk of CD and bread intake might decrease the risk of UC. In Jakosen et al.’s study, whole meal bread consumption was found to be a protective factor for CD, while white bread consumption and cereal cornflake type were found to be risk factors (33).

Dairy products, including milk, yogurt, and cheese, are common components of a Western diet. In the EPIC cohort, the researchers found that dairy product consumption may be associated with a decreased risk of CD (39). Our study also suggested that there might be a negative association between cheese and CD risk.

Several studies have demonstrated that alcohol modulates the immune system in a dose- and time-dependent manner (40, 41). However, in a recent Mendelian randomization analysis conducted by Xia Jiang et al., alcohol intake did not show a causal role in IBD risk (42). In addition, our study did not find any association between alcohol intake and IBD either.

Our research has several significant strengths, out of which the dominant one is the MR design, which is suitable for causal inference. Given the numerous challenges of designing and carrying out RCTs in IBD, an MR study could provide important insights into the associations between specific dietary components and the risk of developing IBD. Furthermore, the food intake factors included in our research, such as processed meat intake, have not previously been investigated in an MR setting. As a result, this study could serve as a model for future research into the relationship between food intake and disease risk.

However, some limitations in this MR study should be observed. First, food intake GWAS remains in its infancy in sample size and could bring compromised statistical power. The limited IV numbers weaken the proportion of phenotypic variance explained. Therefore, the null findings for some associations do not necessarily indicate that food intake has no effect. Second, we only included 17 kinds of food, as other food intakes (i.e., milk intake) do not have enough effective IVs. In addition, one thing should be noted: although only single food items were investigated in our study, these elements may act synergistically or antagonistically as part of a habitual diet (43). The dietary patterns should be studied in MR research to assess their role in CD and UC risk.

In conclusion, we thoroughly examined the potential causal relationship between food intakes and CD and UC. Two types of food intake (poultry intake and cereal intake) were found to increase the risk of CD, and high oily fish intake was associated with UC risk. More research is needed in the future to determine the exact causal relationship and mechanism underlying specific food intakes and IBD.

## Data availability statement

The original contributions presented in the study are included in the article/**Supplementary Material**. Further inquiries can be directed to the corresponding author.

## Author contributions

All authors listed have made a substantial, direct, and intellectual contribution to the work, and approved it for publication.

## Funding

This work was supported by Research foundation of Guangzhou Women and Children’s Medical Center for Clinical Doctor (grant number 1600111).

## Conflict of interest

The authors declare that the research was conducted in the absence of any commercial or financial relationships that could be construed as a potential conflict of interest.

## Publisher’s note

All claims expressed in this article are solely those of the authors and do not necessarily represent those of their affiliated organizations, or those of the publisher, the editors and the reviewers. Any product that may be evaluated in this article, or claim that may be made by its manufacturer, is not guaranteed or endorsed by the publisher.

## References

[1] Abraham C Cho JH. Inflammatory bowel disease. N Engl J Med (2009) 361:2066–78. doi: 10.1056/NEJMra0804647 PMC3491806 19923578

[2] Tracy M Khalili H. You are what you eat? growing evidence that diet influences the risk of inflammatory bowel disease. J Crohns Colitis (2022) 16:1185–6. doi: 10.1093/ecco-jcc/jjac025 PMC9426666 35194635

[3] Lomer MC Thompson RP Powell JJ. Fine and ultrafine particles of the diet: Influence on the mucosal immune response and association with crohn’s disease. Proc Nutr Soc (2002) 61:123–30. doi: 10.1079/pns2001134 12002786

[4] Andersen V Chan S Luben R Khaw KT Olsen A Tjonneland A. Fibre intake and the development of inflammatory bowel disease: A European prospective multi-centre cohort study (EPIC-IBD). J Crohns Colitis (2018) 12:129–36. doi: 10.1093/ecco-jcc/jjx136 PMC5881771 29373726

[5] Maslowski KM Mackay CR. Diet, gut microbiota and immune responses. Nat Immunol (2011) 12:5–9. doi: 10.1038/ni0111-5 21169997

[6] Desai MS Seekatz AM Koropatkin NM Kamada N Hickey CA Wolter M. A dietary fiber-deprived gut microbiota degrades the colonic mucus barrier and enhances pathogen susceptibility. Cell (2016) 167:1339–53. doi: 10.1016/j.cell.2016.10.043 PMC5131798 27863247

[7] Hou JK Abraham B El-Serag H. Dietary intake and risk of developing inflammatory bowel disease: A systematic review of the literature. Am J Gastroenterol (2011) 106:563–73. doi: 10.1038/ajg.2011.44 21468064

[8] West SG Thoemmes F. Campbell’s and rubin’s perspectives on causal inference. Psychol Methods (2010) 15:18–37. doi: 10.1037/a0015917 20230100

[9] Steeger CM Buckley PR Pampel FC Gust CJ Hill KG. Common methodological problems in randomized controlled trials of preventive interventions. Prev Sci (2021) 22:1159–72. doi: 10.1007/s11121-021-01263-2 34176002

[10] Smith GD Ebrahim S. ‘Mendelian randomization’: Can genetic epidemiology contribute to understanding environmental determinants of disease? Int J Epidemiol (2003) 32:1–22. doi: 10.1093/ije/dyg070 12689998

[11] Collins R. What makes UK biobank special? Lancet (2012) 379:1173–4. doi: 10.1016/S0140-6736(12)60404-8 22463865

[12] de Lange KM Moutsianas L Lee JC Lamb CA Luo Y Kennedy NA. Genome-wide association study implicates immune activation of multiple integrin genes in inflammatory bowel disease. Nat Genet (2017) 49:256–61. doi: 10.1038/ng.3760 PMC5289481 28067908

[13] Park S Lee S Kim Y Lee Y Kang MW Kim K. Atrial fibrillation and kidney function: A bidirectional mendelian randomization study. Eur Heart J (2021) 42:2816–23. doi: 10.1093/eurheartj/ehab291 34023889

[14] Staley JR Blackshaw J Kamat MA Ellis S Surendran P Sun BB. PhenoScanner: A database of human genotype-phenotype associations. Bioinformatics (2016) 32:3207–9. doi: 10.1093/bioinformatics/btw373 PMC5048068 27318201

[15] Emdin CA Khera AV Kathiresan S. Mendelian randomization. JAMA (2017) 318:1925–6. doi: 10.1001/jama.2017.17219 29164242

[16] Palmer TM Lawlor DA Harbord RM Sheehan NA Tobias JH Timpson NJ. Using multiple genetic variants as instrumental variables for modifiable risk factors. Stat Methods Med Res (2012) 21:223–42. doi: 10.1177/0962280210394459 PMC3917707 21216802

[17] Bowden J Davey SG Burgess S. Mendelian randomization with invalid instruments: Effect estimation and bias detection through egger regression. Int J Epidemiol (2015) 44:512–25. doi: 10.1093/ije/dyv080 PMC4469799 26050253

[18] Verbanck M Chen CY Neale B Do R. Detection of widespread horizontal pleiotropy in causal relationships inferred from mendelian randomization between complex traits and diseases. Nat Genet (2018) 50:693–8. doi: 10.1038/s41588-018-0099-7 PMC6083837 29686387

[19] Lawlor DA Harbord RM Sterne JA Timpson N Davey SG. Mendelian randomization: Using genes as instruments for making causal inferences in epidemiology. Stat Med (2008) 27:1133–63. doi: 10.1002/sim.3034 17886233

[20] Yavorska OO Burgess S. MendelianRandomization: An r package for performing mendelian randomization analyses using summarized data. Int J Epidemiol (2017) 46:1734–9. doi: 10.1093/ije/dyx034 PMC5510723 28398548

[21] Murdaca G Colombo BM Puppo F. The role of Th17 lymphocytes in the autoimmune and chronic inflammatory diseases. Intern Emerg Med (2011) 6:487–95. doi: 10.1007/s11739-011-0517-7 21258875

[22] Peters V Bolte L Schuttert E Andreu-Sanchez S Dijkstra G Weersma R. Western And carnivorous dietary patterns are associated with greater likelihood of IBD-development in a large prospective population-based cohort. J Crohns Colitis (2021) 16:931–9. doi: 10.1093/ecco-jcc/jjab219 PMC9282880 34864946

[23] Lo CH Khandpur N Rossato SL Lochhead P Lopes EW Burke KE. Ultra-processed foods and risk of crohn’s disease and ulcerative colitis: A prospective cohort study. Clin Gastroenterol Hepatol (2021) 20:e1323–37. doi: 10.1016/j.cgh.2021.08.031 PMC8882700 34461300

[24] Ananthakrishnan AN Khalili H Song M Higuchi LM Richter JM Nimptsch K. High school diet and risk of crohn’s disease and ulcerative colitis. Inflammation Bowel Dis (2015) 21:2311–9. doi: 10.1097/MIB.0000000000000501 PMC4567521 26236952

[25] Lo CH Lochhead P Khalili H Song M Tabung FK Burke KE. Dietary inflammatory potential and risk of crohn’s disease and ulcerative colitis. Gastroenterology (2020) 159:873–83. doi: 10.1053/j.gastro.2020.05.011 PMC7502466 32389666

[26] Ananthakrishnan AN Khalili H Konijeti GG Higuchi LM de Silva P Korzenik JR. A prospective study of long-term intake of dietary fiber and risk of crohn’s disease and ulcerative colitis. Gastroenterology (2013) 145:970–7. doi: 10.1053/j.gastro.2013.07.050 PMC3805714 23912083

[27] Werner T Wagner SJ Martinez I Walter J Chang JS Clavel T. Depletion of luminal iron alters the gut microbiota and prevents crohn’s disease-like ileitis. Gut (2011) 60:325–33. doi: 10.1136/gut.2010.216929 21076126

[28] Fiorucci S Orlandi S Mencarelli A Caliendo G Santagada V Distrutti E. Enhanced activity of a hydrogen sulphide-releasing derivative of mesalamine (ATB-429) in a mouse model of colitis. Br J Pharmacol (2007) 150:996–1002. doi: 10.1038/sj.bjp.0707193 17339831 PMC2013915

[29] Medani M Collins D Docherty NG Baird AW O’Connell PR Winter DC. Emerging role of hydrogen sulfide in colonic physiology and pathophysiology. Inflammation Bowel Dis (2011) 17:1620–5. doi: 10.1002/ibd.21528 21674719

[30] Narula N Wong E Dehghan M Mente A Rangarajan S Lanas F. Association of ultra-processed food intake with risk of inflammatory bowel disease: Prospective cohort study. BMJ (2021) 374:n1554. doi: 10.1136/bmj.n1554 34261638 PMC8279036

[31] Racine A Carbonnel F Chan SS Hart AR Bueno-de-Mesquita HB Oldenburg B. Dietary patterns and risk of inflammatory bowel disease in europe: Results from the EPIC study. Inflammation Bowel Dis (2016) 22:345–54. doi: 10.1097/MIB.0000000000000638 26717318

[32] Vinolo MA Rodrigues HG Nachbar RT Curi R. Regulation of inflammation by short chain fatty acids. Nutrients (2011) 3:858–76. doi: 10.3390/nu3100858 PMC3257741 22254083

[33] Jakobsen C Paerregaard A Munkholm P Wewer V. Environmental factors and risk of developing paediatric inflammatory bowel disease – a population based study 2007-2009. J Crohns Colitis (2013) 7:79–88. doi: 10.1016/j.crohns.2012.05.024 22748696

[34] Roberts CL Keita AV Duncan SH O’Kennedy N Soderholm JD Rhodes JM. Translocation of crohn’s disease escherichia coli across m-cells: Contrasting effects of soluble plant fibres and emulsifiers. Gut (2010) 59:1331–9. doi: 10.1136/gut.2009.195370 PMC2976079 20813719

[35] Spooren CE Pierik MJ Zeegers MP Feskens EJ Masclee AA Jonkers DM. Review article: The association of diet with onset and relapse in patients with inflammatory bowel disease. Aliment Pharmacol Ther (2013) 38:1172–87. doi: 10.1111/apt.12501 24118051

[36] Andersen V Olsen A Carbonnel F Tjonneland A Vogel U. Diet and risk of inflammatory bowel disease. Dig Liver Dis (2012) 44:185–94. doi: 10.1016/j.dld.2011.10.001 22055893

[37] Zhao M Feng R Ben-Horin S Zhuang X Tian Z Li X. Systematic review with meta-analysis: Environmental and dietary differences of inflammatory bowel disease in Eastern and Western populations. Aliment Pharmacol Ther (2022) 55:266–76. doi: 10.1111/apt.16703 34820868

[38] Jantchou P Morois S Clavel-Chapelon F Boutron-Ruault MC Carbonnel F. Animal protein intake and risk of inflammatory bowel disease: The E3N prospective study. Am J Gastroenterol (2010) 105:2195–201. doi: 10.1038/ajg.2010.192 20461067

[39] Opstelten JL Leenders M Dik VK Chan SS van Schaik FD Khaw KT. Dairy products, dietary calcium, and risk of inflammatory bowel disease: Results from a european prospective cohort investigation. Inflammation Bowel Dis (2016) 22:1403–11. doi: 10.1097/MIB.0000000000000798 27120568

[40] Barr T Helms C Grant K Messaoudi I. Opposing effects of alcohol on the immune system. Prog Neuropsychopharmacol Biol Psychiatry (2016) 65:242–51. doi: 10.1016/j.pnpbp.2015.09.001 PMC4911891 26375241

[41] Zhang H Zhu Z Zhang F Meadows GG. Alcohol consumption and antitumor immunity: Dynamic changes from activation to accelerated deterioration of the immune system. Adv Exp Med Biol (2015) 815:313–31. doi: 10.1007/978-3-319-09614-8_18 25427915

[42] Jiang X Zhu Z Manouchehrinia A Olsson T Alfredsson L Kockum I. Alcohol consumption and risk of common autoimmune inflammatory diseases-evidence from a Large-scale genetic analysis totaling 1 million individuals. Front Genet (2021) 12:687745. doi: 10.3389/fgene.2021.687745 34239545 PMC8258244

[43] Hu FB. Dietary pattern analysis: A new direction in nutritional epidemiology. Curr Opin Lipidol (2002) 13:3–9. doi: 10.1097/00041433-200202000-00002 11790957

