# Supplementary figures and images for: Mendelian randomization analysis reveals causal effects of food intakes on inflammatory bowel disease risk
Source: Front Immunol. 2022 Sep 22;13:911631. doi: 10.3389/fimmu.2022.911631 (PMC9536736; doi:10.3389/fimmu.2022.911631)

A

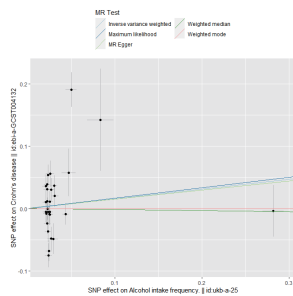

B

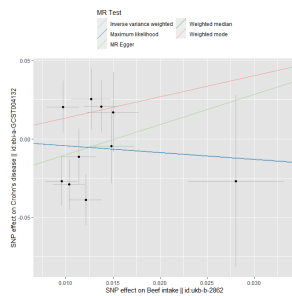

C

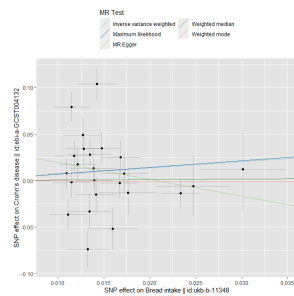

D

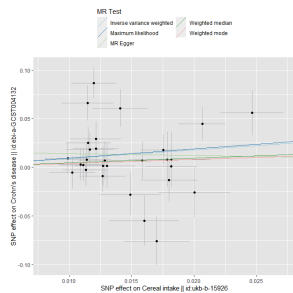

E

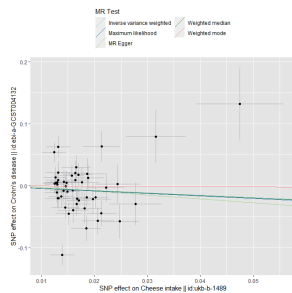

F

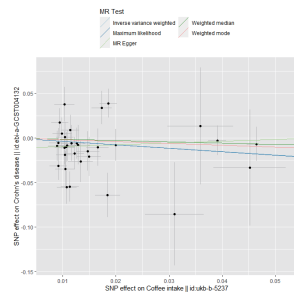

G

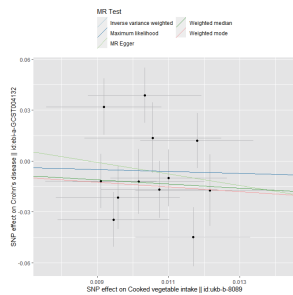

H

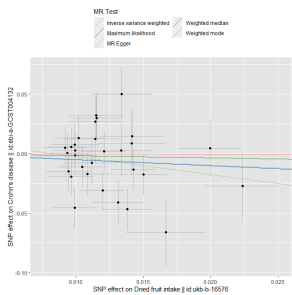

I

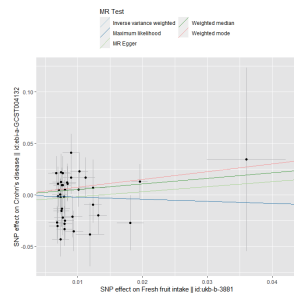

J

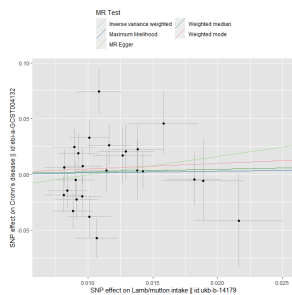

K

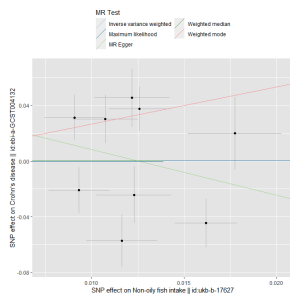

L

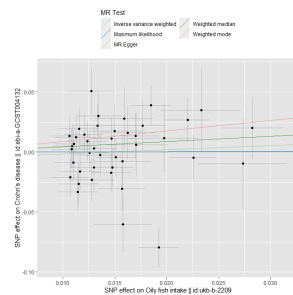

M

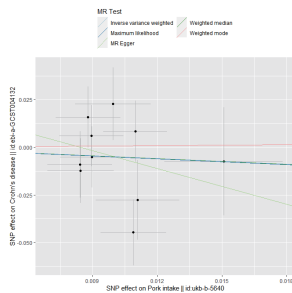

N

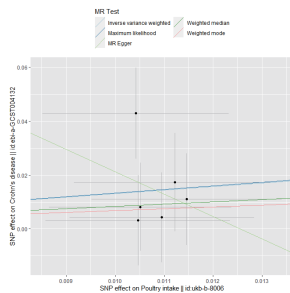

O

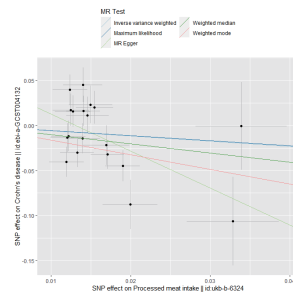

P

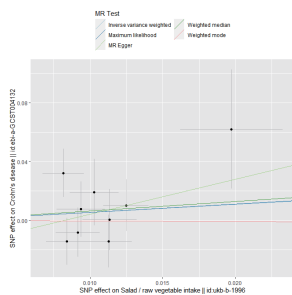

Q

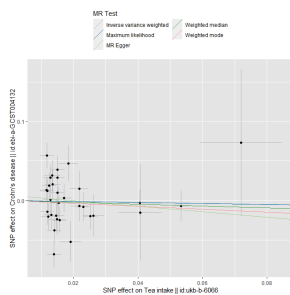

Supplement: Supplementary file 1 [file Image_1.pdf]

A

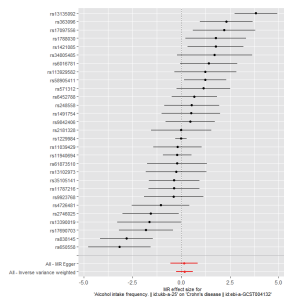

B

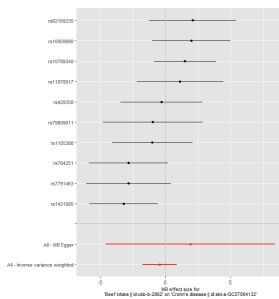

C

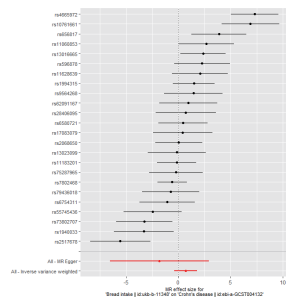

D

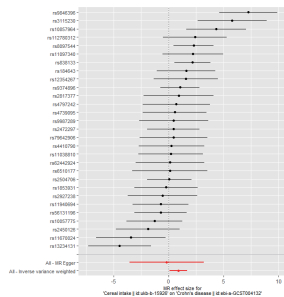

E

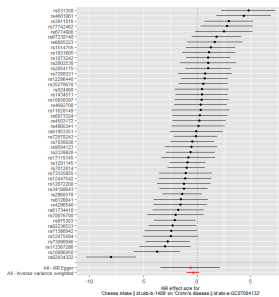

F

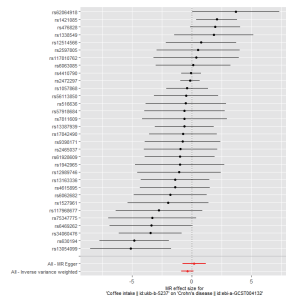

G

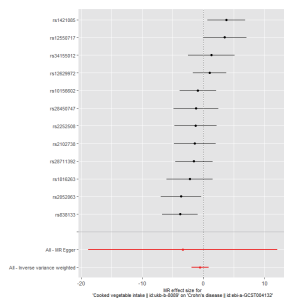

H

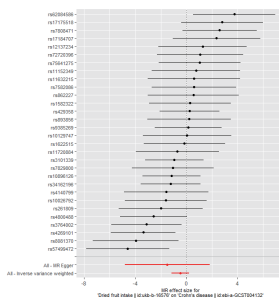

I

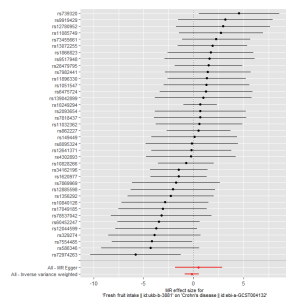

J

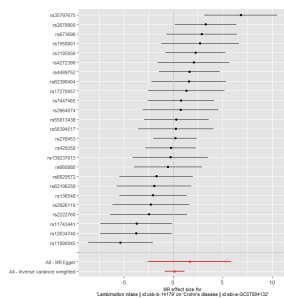

K

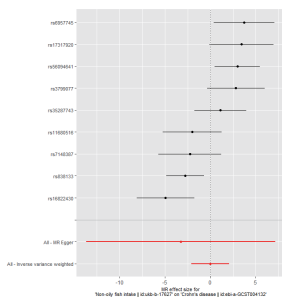

L

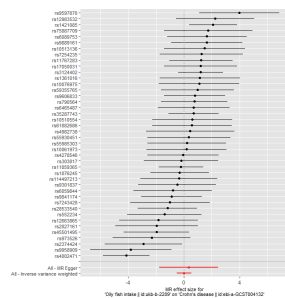

M

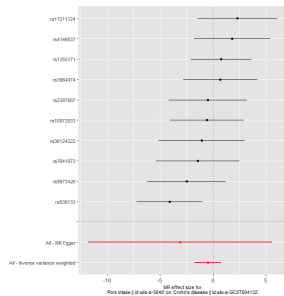

N

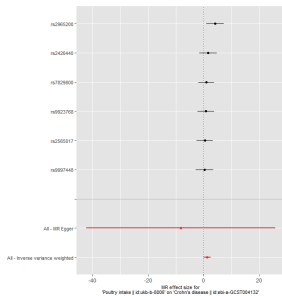

O

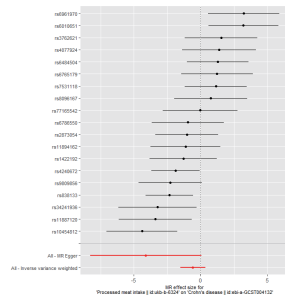

P

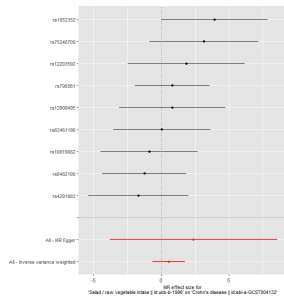

Q

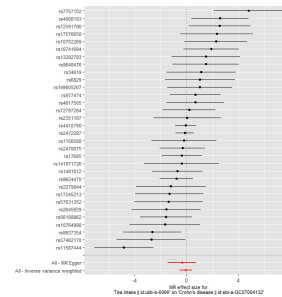

Supplement: Supplementary file 2 [file Image_2.pdf]

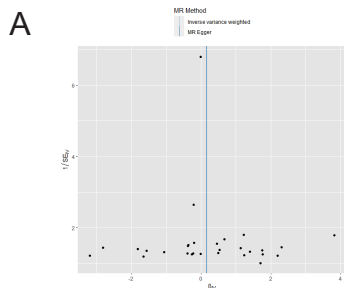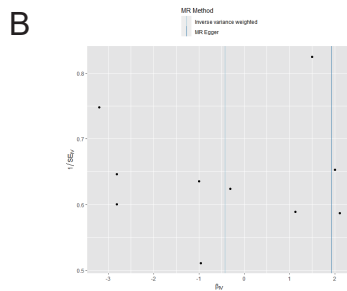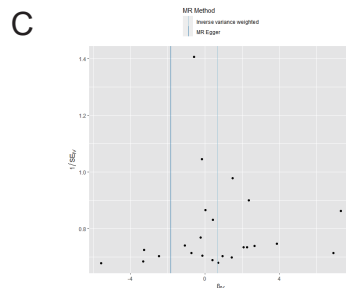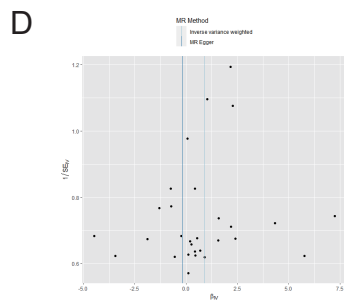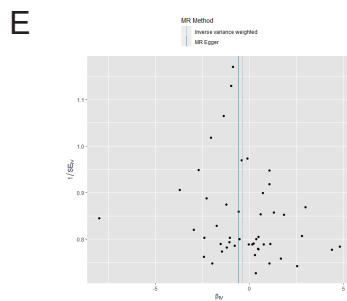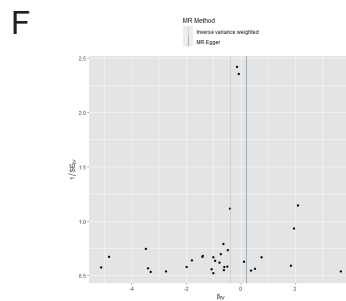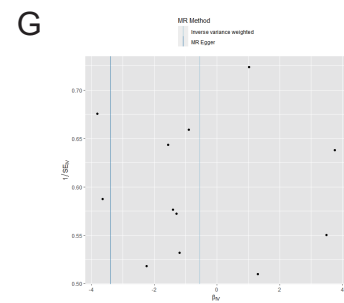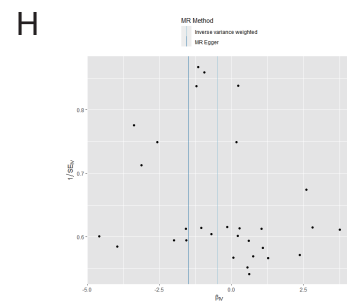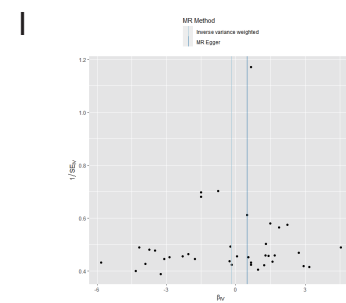

J

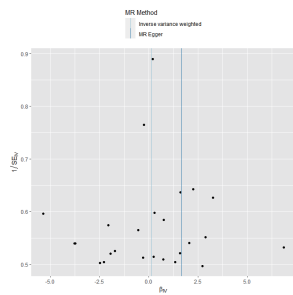

K

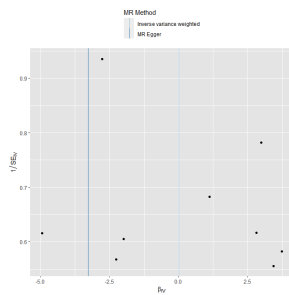

L

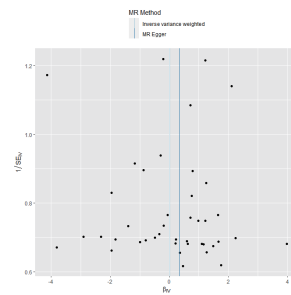

M

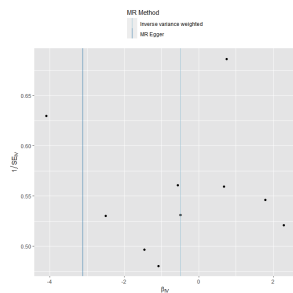

N

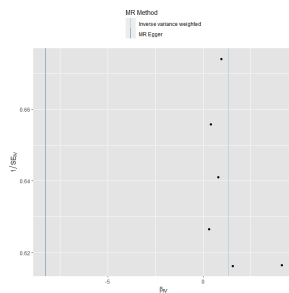

O

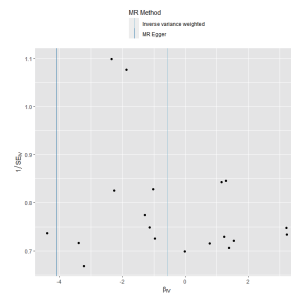

P

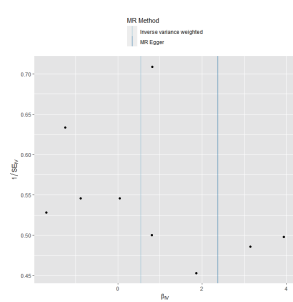

Q

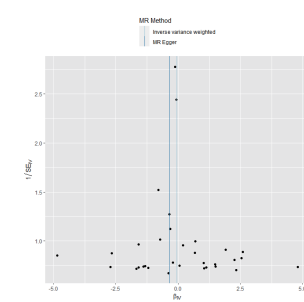

Supplement: Supplementary file 3 [file Image_3.pdf]

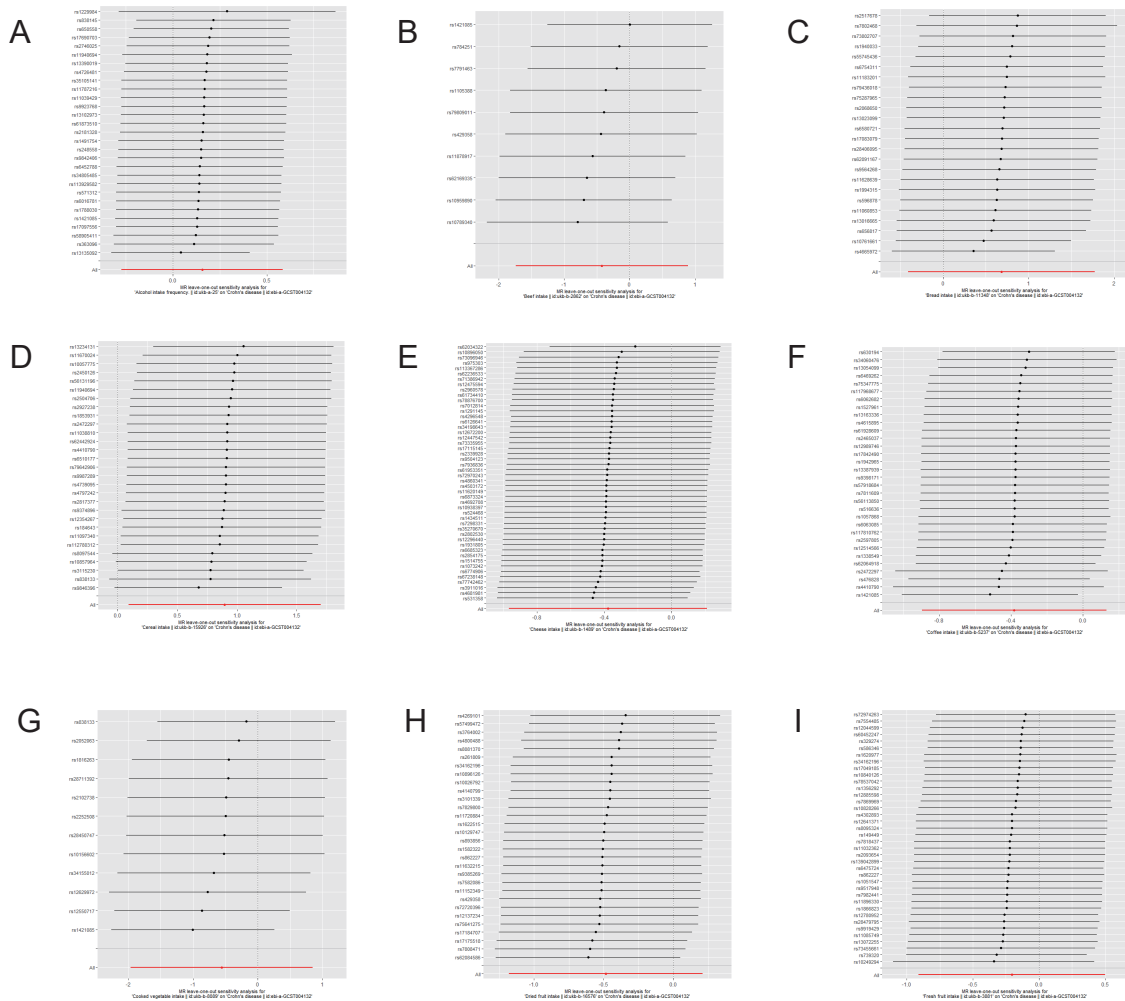

Supplement: Supplementary file 4 [file Image_4.pdf]

A

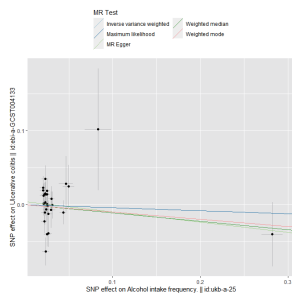

B

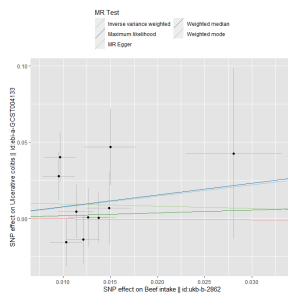

C

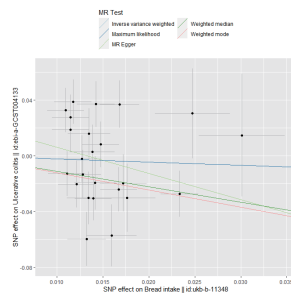

D

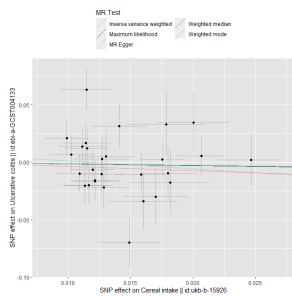

E

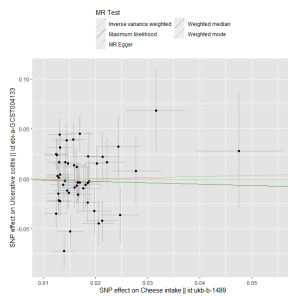

F

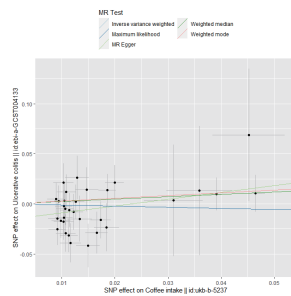

G

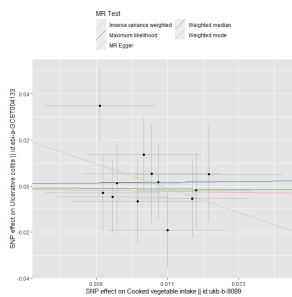

H

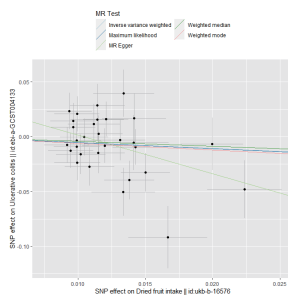

I

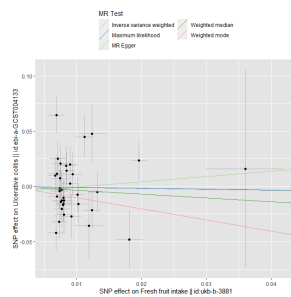

J

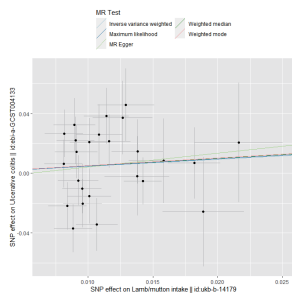

K

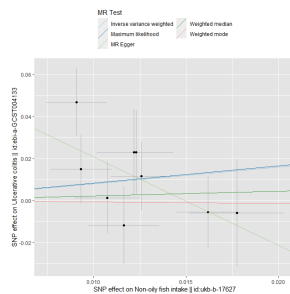

L

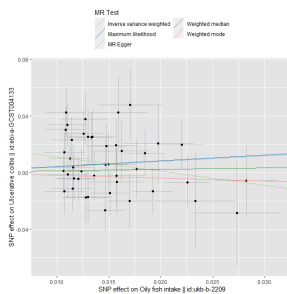

M

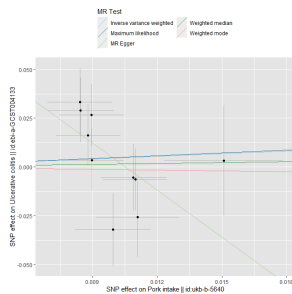

N

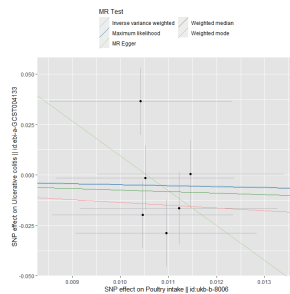

O

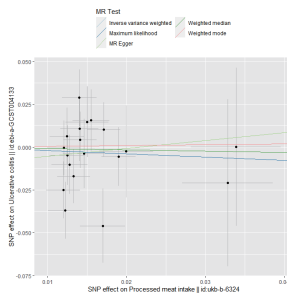

P

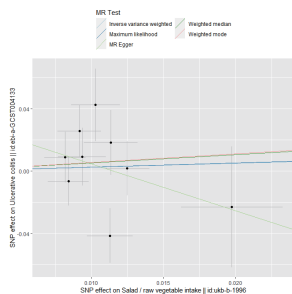

Q

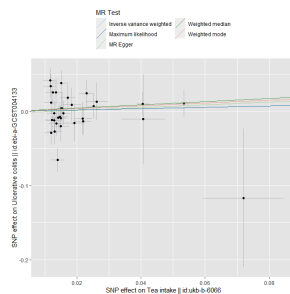

Supplement: Supplementary file 5 [file Image_5.pdf]

A

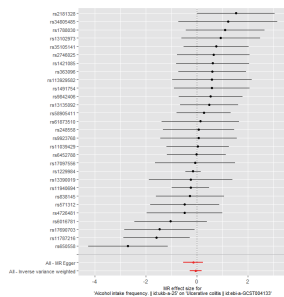

B

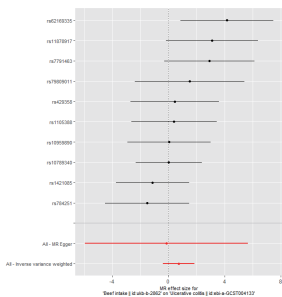

C

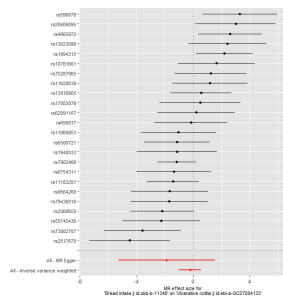

D

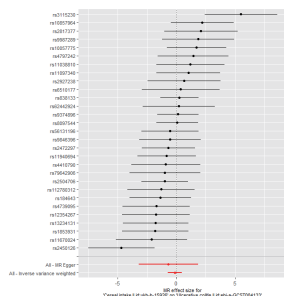

E

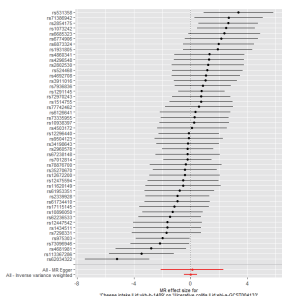

F

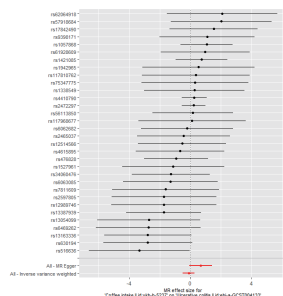

G

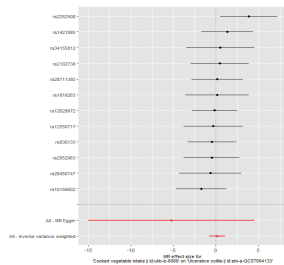

H

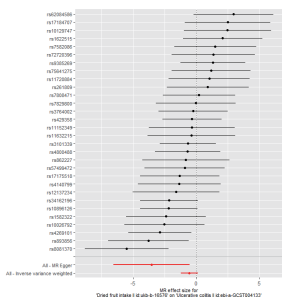

I

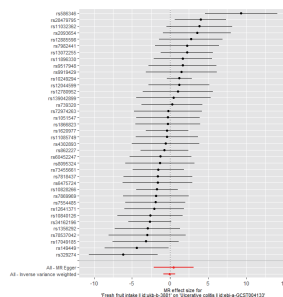

Supplement: Supplementary file 6 [file Image_6.pdf]

A

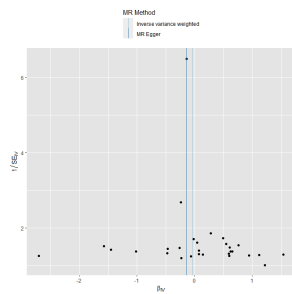

B

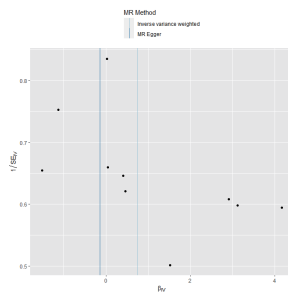

C

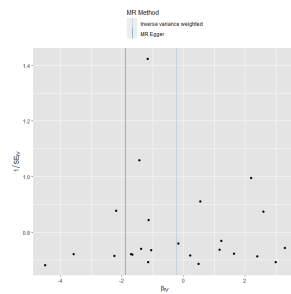

D

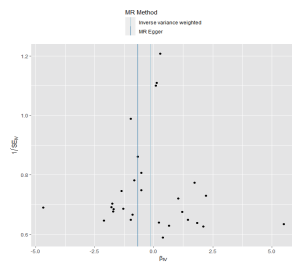

E

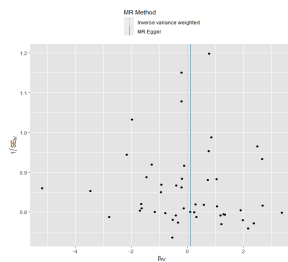

F

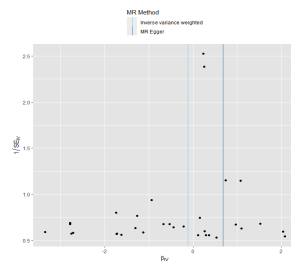

G

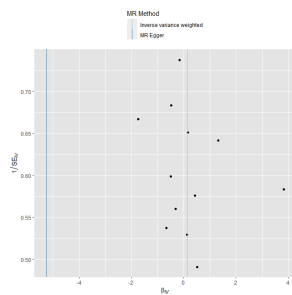

H

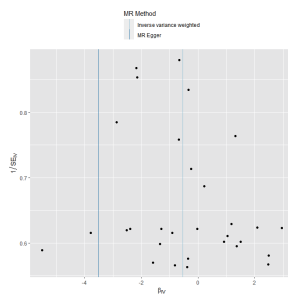

I

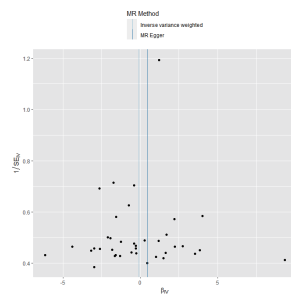

J

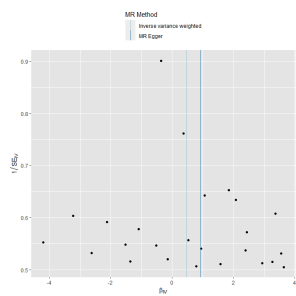

K

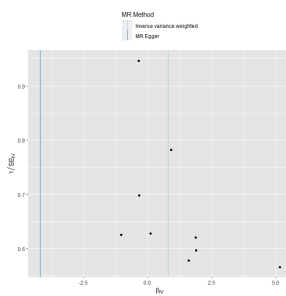

L

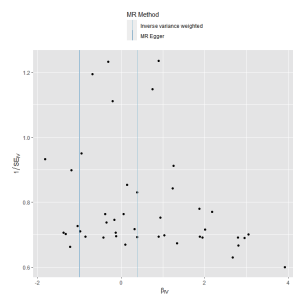

M

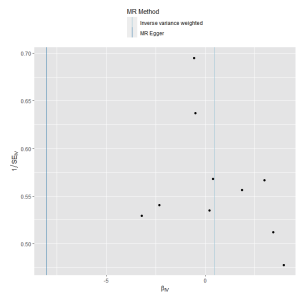

N

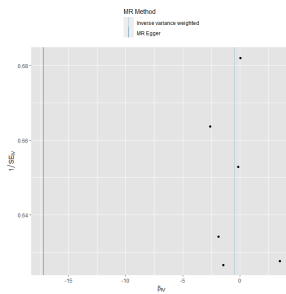

O

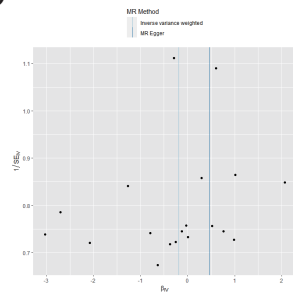

P

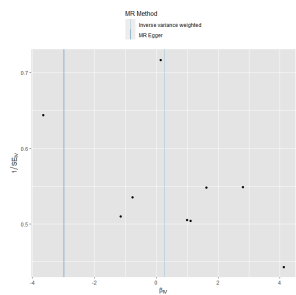

Q

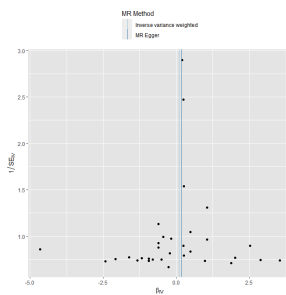

Supplement: Supplementary file 7 [file Image_7.pdf]
