# Supplementary material for: Mendelian randomization analysis reveals causal effects of food intakes on inflammatory bowel disease risk
Source: Front Immunol. 2022 Sep 22;13:911631. doi: 10.3389/fimmu.2022.911631 (PMC9536736; doi:10.3389/fimmu.2022.911631)
Supplement: Supplementary file 8 [file Image_8.pdf]

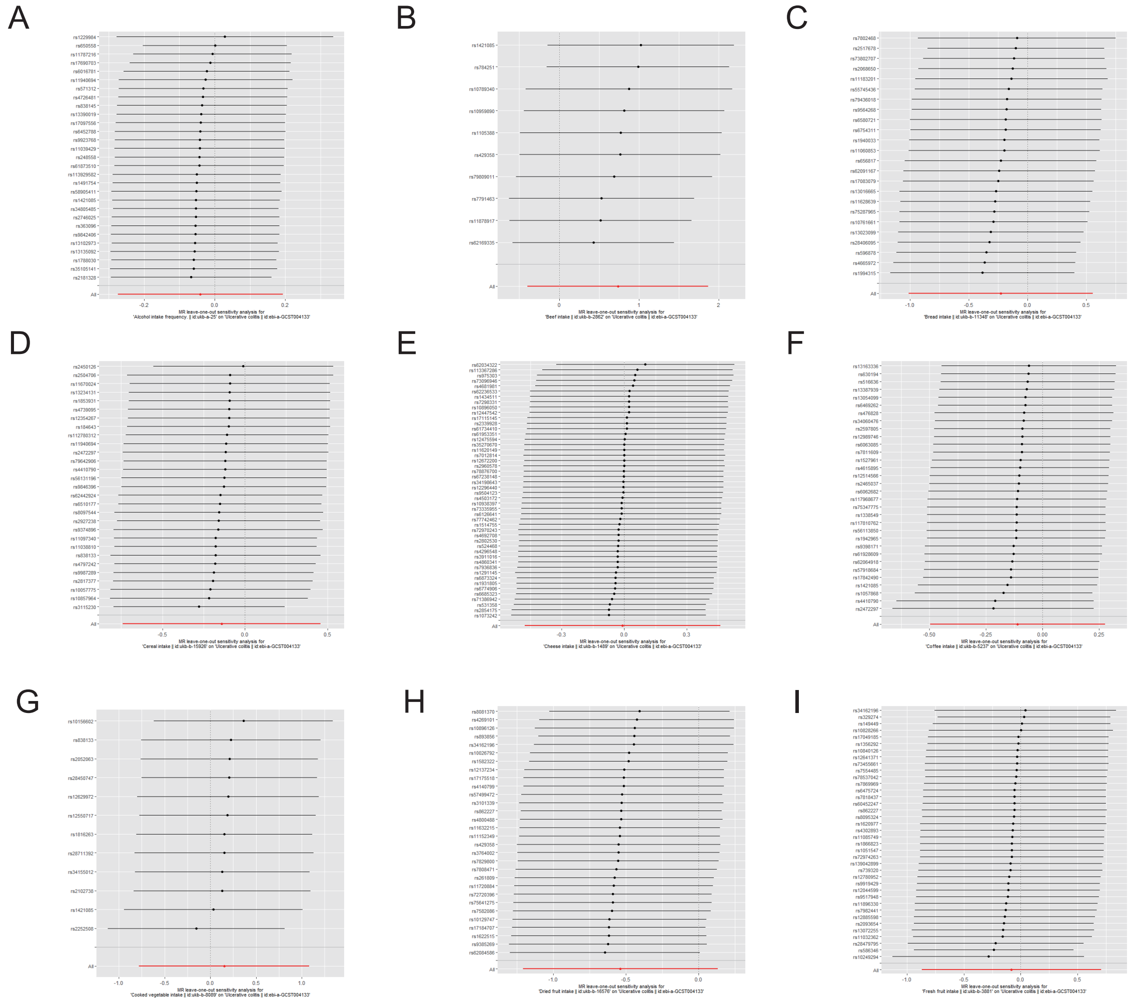
A
MR leave-one-out sensitivity analysis for
'Alcohol intake frequency. || id:ukb-a-25' on 'Ulcerative colitis || id:ebi-a-GCST004133'
B
MR leave-one-out sensitivity analysis for
'Beef intake || id:ukb-b-2862' on 'Ulcerative colitis || id:ebi-a-GCST004133'
C
MR leave-one-out sensitivity analysis for
'Bread intake || id:ukb-b-11348' on 'Ulcerative colitis || id:ebi-a-GCST004133'
D
MR leave-one-out sensitivity analysis for
'Cereal intake || id:ukb-b-15926' on 'Ulcerative colitis || id:ebi-a-GCST004133'
E
MR leave-one-out sensitivity analysis for
'Cheese intake || id:ukb-b-1489' on 'Ulcerative colitis || id:ebi-a-GCST004133'
F
MR leave-one-out sensitivity analysis for
'Coffee intake || id:ukb-b-5237' on 'Ulcerative colitis || id:ebi-a-GCST004133'
G
MR leave-one-out sensitivity analysis for
'Cooked vegetable intake || id:ukb-b-8089' on 'Ulcerative colitis || id:ebi-a-GCST004133'
H
MR leave-one-out sensitivity analysis for
'Dried fruit intake || id:ukb-b-16576' on 'Ulcerative colitis || id:ebi-a-GCST004133'
I
MR leave-one-out sensitivity analysis for
'Fresh fruit intake || id:ukb-b-3881' on 'Ulcerative colitis || id:ebi-a-GCST004133'

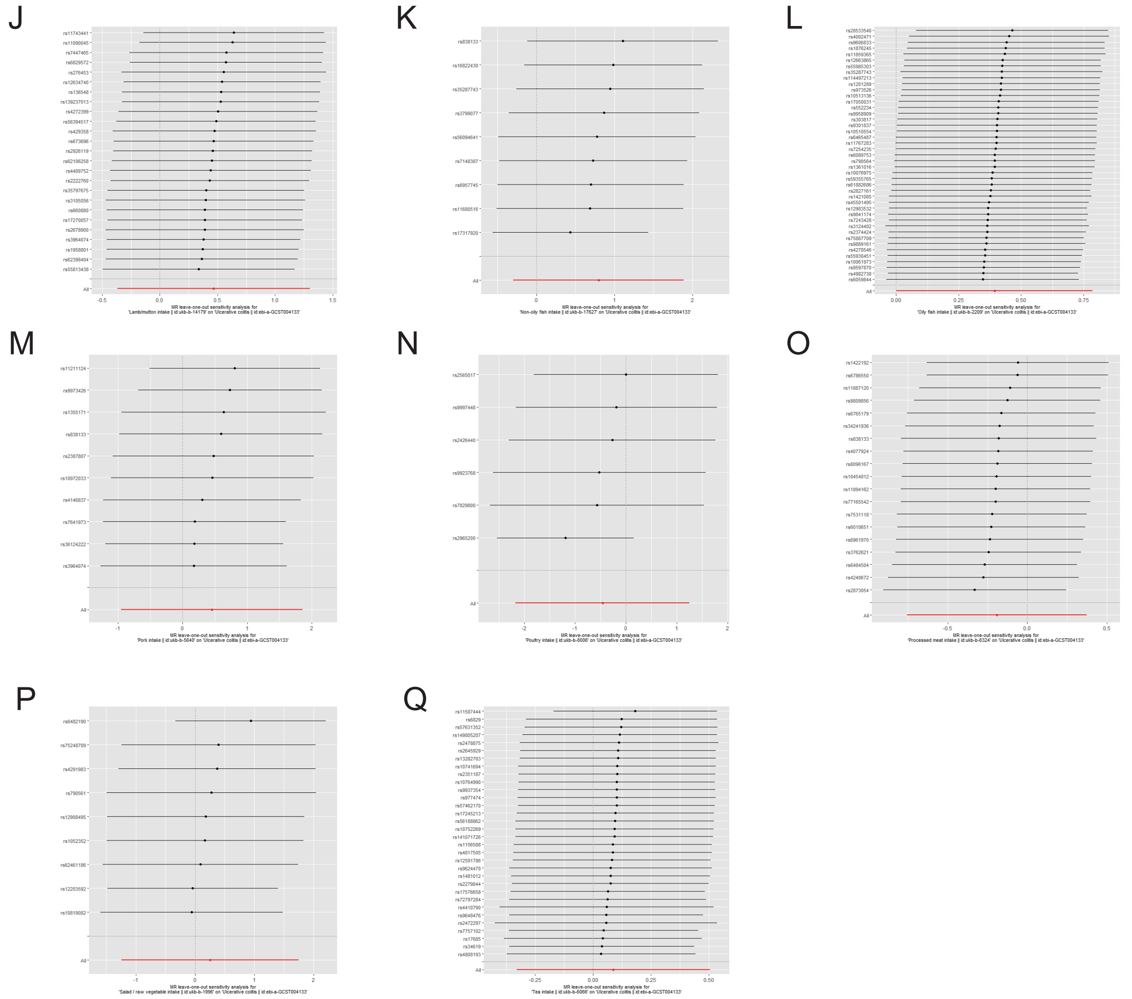
J
MR leave-one-out sensitivity analysis for
'Lamb/mutton intake || id:ukb-b-14179' on 'Ulcerative colitis || id:ebi-a-GCST004133'
K
MR leave-one-out sensitivity analysis for
'Non-oily fish intake || id:ukb-b-17627' on 'Ulcerative colitis || id:ebi-a-GCST004133'
L
MR leave-one-out sensitivity analysis for
'Oily fish intake || id:ukb-b-2209' on 'Ulcerative colitis || id:ebi-a-GCST004133'
M
MR leave-one-out sensitivity analysis for
'Pork intake || id:ukb-b-5640' on 'Ulcerative colitis || id:ebi-a-GCST004133'
N
MR leave-one-out sensitivity analysis for
'Poultry intake || id:ukb-b-8006' on 'Ulcerative colitis || id:ebi-a-GCST004133'
O
MR leave-one-out sensitivity analysis for
'Processed meat intake || id:ukb-b-6324' on 'Ulcerative colitis || id:ebi-a-GCST004133'
P
MR leave-one-out sensitivity analysis for
'Salad / raw vegetable intake || id:ukb-b-1996' on 'Ulcerative colitis || id:ebi-a-GCST004133'
Q
MR leave-one-out sensitivity analysis for
'Tea intake || id:ukb-b-6066' on 'Ulcerative colitis || id:ebi-a-GCST004133'
